# Food Components in Health Promotion and Disease Prevention

**DOI:** 10.3390/foods12244401

**Published:** 2023-12-07

**Authors:** Annalisa Chiavaroli, Luigi Brunetti

**Affiliations:** Department of Pharmacy, G. d’Annunzio University of Chieti-Pescara, Via dei Vestini 31, 66013 Chieti, Italy; luigi.brunetti@unich.it

In recent years, more plant-based sources of functional foods have been shown to be effective in preventing, reducing, and treating chronic inflammatory and metabolic diseases, and promoting health [1]. There is a great deal of interest in food with reference to bioactive compounds present in natural products, such as vegetables and fruits extracted from wastes and by-products, since they can exert various beneficial effects by virtue of their anti-inflammatory and antioxidant properties [2].

This Special Issue contains thirteen papers, which explore the possible protective role of foods, their components, and natural antioxidant compounds, together with an accurate evaluation of qualitative and quantitative chemical composition of foods and their ingredients. The contributions covered a variety of chronic inflammatory and metabolic disorders such as cardiovascular disease, metabolic syndrome and ulcerative colitis, exploiting different experimental approaches, as well as attracting reviews.

Interestingly, Recinella’s article (contribution 1) studied the protective effects of aged black garlic water extract alone or in association with multivitamins on mouse heart specimens exposed to *E. coli* lipopolysaccharide. The authors showed that the extract exerted beneficial actions on isolated samples, as corroborated by the inhibitory activities on multiple pro-inflammatory and oxidative stress-related biomarkers. The protective effects could be related to the high content in the extract of two polyphenolic compounds known for their preventive action at the cardiovascular level: catechin and gallic acid [3,4,5].

In this context, the effect of *Aronia melanocarpa* fruit juice supplementation was evaluated on age-related coronary arteries by Daskalova and collaborators (contribution 2). The results highlighted that the treatment had a positive impact in the experimental model used, supporting the potential use of antioxidant-rich foods in age-related diseases. In agreement, black chokeberry (*Aronia melanocarpa* L.) intake showed effectiveness in recent studies in attenuating obesity-induced colonic inflammation, in preventing gut microbiota dysbiosis, and in improving intestinal lesions in inflammatory bowel diseases (IBDs) [6,7,8]. Chokeberry fruits, due to their high polyphenol content, could soon be recommended to prevent and treat many metabolic disorders [9].

In addition, Alonso-Bastida and collaborators (contribution 3) investigated glycemic variation in physically active persons consuming different macronutrients; their findings revealed the beneficial effects in glucose levels of eating healthy foods before carbohydrates. Further studies are needed to develop alternative solutions using a correct sequence of food intake for metabolic and neurodegenerative diseases [10].

Another interesting study (contribution 4) focused on sodium levels in Malaysian street food and found that processed foods present in the main street food dishes contain high quantities of sodium, higher than the recommended daily dose. It is certainly necessary to reduce the salt content in these foods and suggest replacements with products low in sodium and simultaneously rich in potassium, as demonstrated by Marklund and collaborators [11].

Based on these results, a subsequent study (contribution 5) was conducted to better understand the correlation between correct eating behaviors and consumption of dietary fiber (DF), associating them with frequency of food label reading with reference to DF. The findings showed that greater awareness and education of the population on the accurate reading of food labels, evaluating the presence of dietary fiber, encourages consumers to purchase increasingly healthier foods [12].

Furthermore, there is an ever-increasing interest in plant extracts, including tea leaf extracts, which act as inhibitors of pathogens. Recently, Liu and collaborators (contribution 6) examined the antibacterial activity of four varieties of tea extracts, highlighting green tea extracts as the most effective against a variety of Gram-positive and Gram-negative bacteria, attributable to the interaction of catechin with the bacterial cell membrane. In this context, various in vitro studies of antibacterial effect exerted by green tea catechin were reported [13,14].

Moreover, garlic extracts have also proven effective in IBD as attested by the study by Recinella and collaborators (contribution 7): both tested extracts exerted protective effects in the colon probably due to the high presence of catechin as also confirmed by other studies improving the potential role of this natural compound as a therapeutic target [15].

Additionally, a further study investigated the composition, possible anti-tumor effects, and molecular mechanisms of extracts from a mushroom (contribution 8). The findings demonstrated that *L. hatsudake* extracts were effective at both inducing apoptosis and arresting the proliferation of cancer cells and further validate the effectiveness of mushrooms and the possible use of their bioactive ingredients against cancer [16,17].

Among the articles, there is a study (contribution 9) on gamma aminobutyric acid-enriched fermented oyster (Crassostrea gigas) extract (FO) efficacy in activating osteoblast differentiation and bone formation; the obtained results confirmed previous data and proposed a future FO use to improve bone health [18].

Interestingly, Matisek and collaborators (contribution 10) studied the potential neuroprotective effects of bioactive compounds present in Beetroot/Carrot Juice in neurodegenerative diseases showing that the drink induced beneficial actions attributable to the activity of natural ingredients with an increase in intracellular Ca^2+^ in neurons, preventing the toxic effects of heavy metals and laying the foundation for future studies on the neuroprotective effects of bioactive compounds [19].

In a review (contribution 11), the authors focused their attention on ellagitannins (ETs) present in many plants and responsible for beneficial health effects on various diseases. They concentrated both on highlighting the most important and innovative characteristics exerted by hydrolysable tannins and gut microbiota-derived metabolites as well as examining their protective activities on the gut–brain axis. In addition, numerous studies confirmed protective actions exerted by ETs on several pathologies including various forms of cancer and toxic compounds. However, further studies are certainly necessary to better understand the potential applications and the toxicological and pharmacological profile of these substances [20,21,22].

Metabolic syndrome is the combination of conditions that together increase the risk of coronary heart disease, diabetes, stroke, and other serious diseases. Eating habits and lifestyle demonstrate a huge impact on the development of metabolic syndrome. Among natural products, there are several bioactive compounds that regulate lipid and carbohydrate metabolism and improve digestion in the human body. In their review, Kania-Dobrowolska and collaborators (contribution 12) focused on dandelion, an edible plant rich in active compounds effective in the treatment and prevention of metabolic syndrome. Several studies showed that dandelion’s extracts can exert multidirectional effects revealing both hypolipidemic and hypoglycemic actions and antiobesity, antioxidant, and antiplatelet activity, corroborating its possible use in the treatment of diabetes and cardiovascular diseases. However, further research is needed to investigate the mechanism of action, safety, and biological activity [23].

Finally, in another review (contribution 13), an objective overview was provided on variations in tumor necrosis factor-alpha levels in subjects with different body mass index and on the effectiveness of a diet composed of different foods and products containing mixed bioactive compounds in overweight/obese subjects. There was no clear correlation between the responses of this cytokine and changes in body weight. According to the authors, it is necessary to use new methods that allow us to consider TNF-α as a biomarker of response to diet [24].

In summary, all research articles and reviews in this Special Issue provide new insights to further investigate the role of foods and natural antioxidant compounds in the management and prevention of chronic inflammatory and metabolic diseases.
